# Physiological and Growth Responses of Potato (*Solanum Tuberosum* L.) to Air Temperature and Relative Humidity under Soil Water Deficits

**DOI:** 10.3390/plants11091126

**Published:** 2022-04-21

**Authors:** Peng Zhang, Xin Yang, Kiril Manevski, Shenglan Li, Zhenhua Wei, Mathias Neumann Andersen, Fulai Liu

**Affiliations:** 1 Key Laboratory of Mollisols Agroecology, Northeast Institute of Geography and Agroecology, Chinese Academy of Sciences, Changchun 130102, China; bhpeng@163.com; 2 Department of Plant and Environmental Science, Faculty of Science, University of Copenhagen, Højbakkegaard Alle 13, 2630 Taastrup, Denmark; yangxin20150303@163.com (X.Y.); 200034@nuist.edu.cn (S.L.); 3 Key Laboratory of Agricultural Soil and Water Engineering in Arid and Semiarid Areas, Ministry of Education, Northwest A&F University, Yangling 712100, China; hnpdswzh@163.com; 4 Department of Agroecology, Aarhus University, Blichers Allé 20, 8830 Tjele, Denmark; kiril.manevski@agro.au.dk (K.M.); mathiasn.andersen@agrsci.dk (M.N.A.); 5 Institute of Facility Agriculture, Guangdong Academy of Agricultural Sciences, Guangzhou 510640, China; 6 Sino-Danish Center for Education and Research, University of Chinese Academy of Sciences, 380 Huaibeizhuang, Beijing 101400, China

**Keywords:** potato, vapor pressure deficit, soil drying, gas exchange, water use efficiency

## Abstract

Drought stress often occurs concurrently with heat stress, yet the interacting effect of high vapor pressure deficit (VPD) and soil drying on the physiology of potato plants remains poorly understood. This study aimed to investigate the physiological and growth responses of potatoes to progressive soil drying under varied VPDs. Potato plants were grown either in four separate climate-controlled greenhouse cells with different VPD levels (viz., 0.70, 1.06, 1.40, and 2.12 kPa, respectively) or under a rainout shelter in the field. The VPD of each greenhouse cell was caused by two air temperature levels (23 and 30 °C) combined with two relative humidity levels (50 and 70%), and the VPD of the field was natural conditions. Irrigation treatments were commenced three or four weeks after planting in greenhouse cells or fields, respectively. The results indicated that soil water deficits limited leaf gas exchange and shoot dry matter (*DM_shoot_*) of plants while increasing the concentration of abscisic acid (ABA) in the leaf and xylem, as well as water use efficiency (*WUE*) across all VPD levels. High VPD decreased stomatal conductance (*g_s_*) but increased transpiration rate (*T_r_*). High VPD increased the threshold of soil water for *T_r_* began to decrease, while the soil water threshold for *g_s_* depended on temperature due to the varied ABA response to temperature. High VPD decreased leaf water potential, leaf area, and *DM_shoot_*, which exacerbated the inhibition of soil drying to plant growth. Across the well-watered plants in both experiments, negative linear relationships of *g_s_* and *WUE* to VPD and positive linear relations between *T_r_* and VPD were found. The results provide some novel information for developing mechanistic models simulating crop *WUE* and improving irrigation scheduling in future arid climates.

## 1. Introduction

The mean air temperature has increased 0.6 °C globally over the past century, and it is predicted to rise by about 3.2 °C by 2100 [1]. Climate change also predicts an increased frequency of extreme drought episodes and associated soil water deficits in many regions [2,3]. Soil water deficit is an important limiting factor for crop yield and quality [4,5]. On the other hand, vapor pressure deficit (VPD) is the driving force for transpiration and, therefore, affects a range of processes in the plant [6]. Reduced air relative humidity at a given temperature increases VPD, which often enhances the transpiration rate (*T_r_*), increases the water consumption, and reduces the plant water use efficiency (*WUE*) [7]. It also limits plant growth due to the restrictions of the stomatal aperture and limitations of the photosynthetic rate (*A_n_*) [8]. However, the interactions between soil water deficit and VPD, and the plant response mechanisms to these two abiotic stresses, are complex and remain largely elusive.

Stomata are superficial leaf pores, and their amount and aperture regulate the gas exchanges between the leaf interior and the atmosphere, thereby influencing plant carbon assimilation and *WUE* [9]. Under soil drying conditions, plant roots generate chemical signals with hormones, primarily abscisic acid (ABA), which are transported via the xylem to the leaf to trigger stomatal closure, thereby reducing stomatal conductance (*g_s_*), and this is particularly evident in potatoes [10,11,12]. Potato (*Solanum tuberosum* L.) is one of the organical crops to be widely grown in Europe, e.g., Denmark and Italy, and is also a typical food crop of many countries in the world [13,14]. However, the growth and production of potatoes have been severely limited in arid and/or high-temperature regions due to it being a drought-sensitive crop that closes its stomata at moderate soil water deficits [15,16]. High temperature directly affects the carbon metabolism and reduces *A_n_* by stomatal and non-stomatal limitations, consequently decreasing the accumulation of assimilation. In this regard, “stomatal limitations” refers to photosynthesis constraints by stomata, which is useful for ecosystem and land-surface modeling studies, whereas “diffusional limitations” explains metabolic effects on photosynthesis, including effects on the electron transport chain and the excessive production of reactive oxygen species [17]. In addition, stomatal behavior is correlated with changes in both chemical and hydraulic signals; however, studies have demonstrated that changes in ABA occurred prior to any change in leaf water status and that the chemical signaling prevails [18]. The assimilate partitioning to tubers could also be reduced significantly under high temperature, resulting in a low tuber yield and quality [19].

*T_r_* and *g_s_* are the two major physiological parameters of plants responding to increased VPD due to reduced relative humidity and/or high temperature [20,21]. Low relative humidity (implying high VPD) reduces the turgor of guard cells and decreases *g_s_* while increasing *T_r_*, which, in turn, could reduce *A_n_* [22,23]. Thus, enhanced evaporative demand and decreased carbon assimilation caused by the combination of high temperature and/or low relative humidity, i.e., high VPD, ‘double-stress’ the plant and negatively affect its growth and *WUE* [24]. On the other hand, Nejad and Meeteren (2003) found that plants developed under high relative humidity have a poor ability to control water loss as compared to those grown under low humidity, attributed to dysfunction of stomata (i.e., larger stomatal pores) and increased cuticular permeability [25]. In addition, the high relative humidity would also result in reduced sensitivity of stomata to soil water deficits and leaf water potential changes, causing excessive water loss and desiccation of the leaves, especially under co-occurrence of hot and humid conditions [26].

Plants encounter a variety of abiotic stresses in the process of growth and development. However, previous studies focused mainly on single soil water deficits or high VPD, and little attention was paid to the interaction effects between soil drying and high VPD. Therefore, the study here aimed to explore the physiological and growth responses of potato plants to the co-occurrence of soil drying and high VPD. Such information is essential for developing mechanistic models simulating *g_s_* and *WUE* of potato plants grown in future climate change scenarios for a better irrigation scheduling of the crop. It was hypothesized that the effect of soil water deficits on crop *WUE* is VPD dependent, and high VPD would override the positive effect of moderate soil water deficits decreasing the *WUE*. Furthermore, the objective of this work was threefold: (i) to examine the effects of high temperature and/or low relative humidity (i.e., elevated VPD) on leaf gas exchange of potato plants under soil water deficits; (ii) to assess the response of potato plants to soil water deficit in combination with the dynamic changes of temperature and relative humidity; (iii) to test for a common relationship of *g_s_*, *T_r_*, and *WUE* to VPD under both controlled (greenhouse) and fluctuated (field) environments.

## 2. Results

### 2.1. Leaf Gas Exchange and Chemical Signals

**Figure 1:**
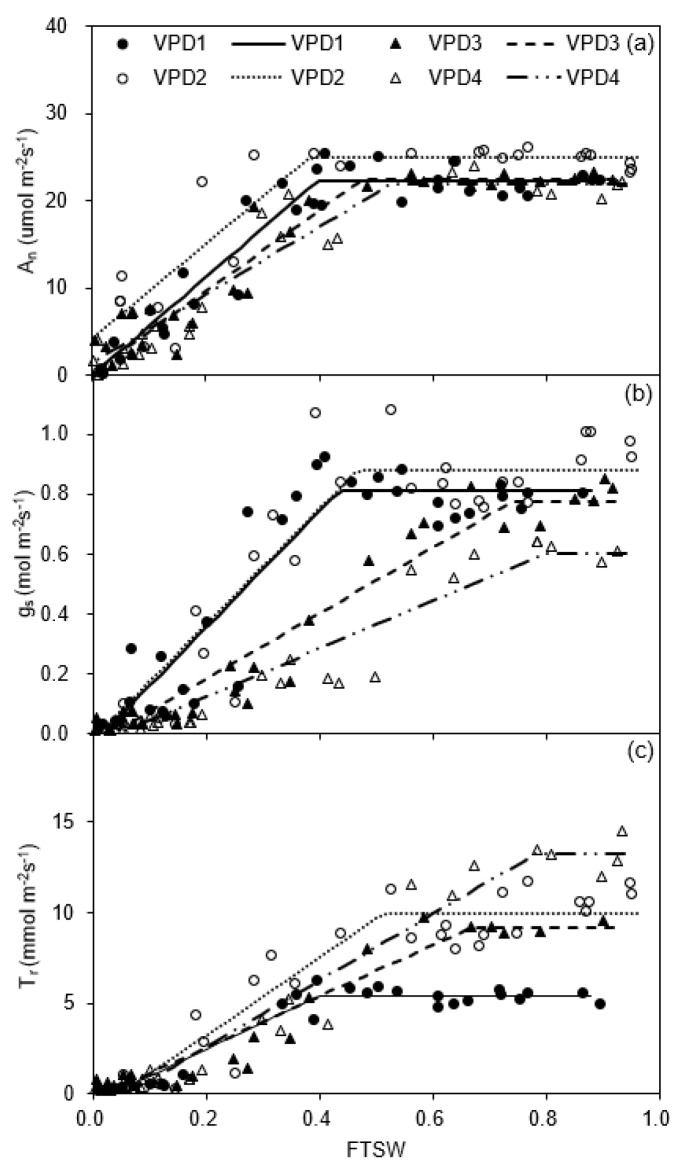
Changes in net photosynthetic rate (*A_n_*; (**a**)), stomatal conductance (*g_s_*; (**b**)) and transpiration rate (*T_r_*; (**c**)) of potato plants grown under different vapor pressure deficits (VPD) in the greenhouse cells during progressive soil drying. VPD1, normal temperature and high relative humidity; VPD2, high temperature and relative humidity; VPD3, normal temperature and low relative humidity; VPD4, high temperature and low relative humidity, the same as below.

For the greenhouse experiment, the linear plateau analysis showed a slightly higher *A_n max_* (24.94 mol m^−2^ s^−1^) for potato plants grown at high temperature and relative humidity (VPD2) than the other VPDs at the onset of soil drying, when *FTSW* > 0.38 (*C_A_*), though without significant difference in *A_n max_* and *C_A_* between the four VPD treatments (Figure 1a; Table 1).

At the onset of soil drying, *g_s_* of the plants grown at high temperature was lower than for normal temperature under low relative humidity; *g_s_* of plants grown under low relative humidity was also lower than for high humidity at high temperature. The *g_s max_* of plants grown under high temperature and low relative humidity (VPD4) was the lowest as compared to other VPD treatments. After imposing drought stress on the plants, *g_s_* at normal and high temperatures under high relative humidity decreased rapidly when the *FTSW* threshold (*C_g_*) dropped to 0.43 and 0.47, respectively, which was significantly lower than those under low relative humidity, i.e., 0.74 and 0.80 at normal and high temperature, respectively (Figure 1b; Table 1).

At a given temperature, plants grown under high relative humidity had a significantly higher *T_r_* compared to those grown under low relative humidity. The *T_r max_* of plants grown under either low or high relative humidity at normal temperature was 38 and 54% lower than those grown at high temperature, respectively. In contrast, the *FTSW* threshold (*C_T_*) at which *T_r_* started to decrease depended on relative humidity. Plants grown under high relative humidity at either temperature have a significantly lower *C_T_* than those grown under low relative humidity (Figure 1c; Table 1).

**Figure 2:**
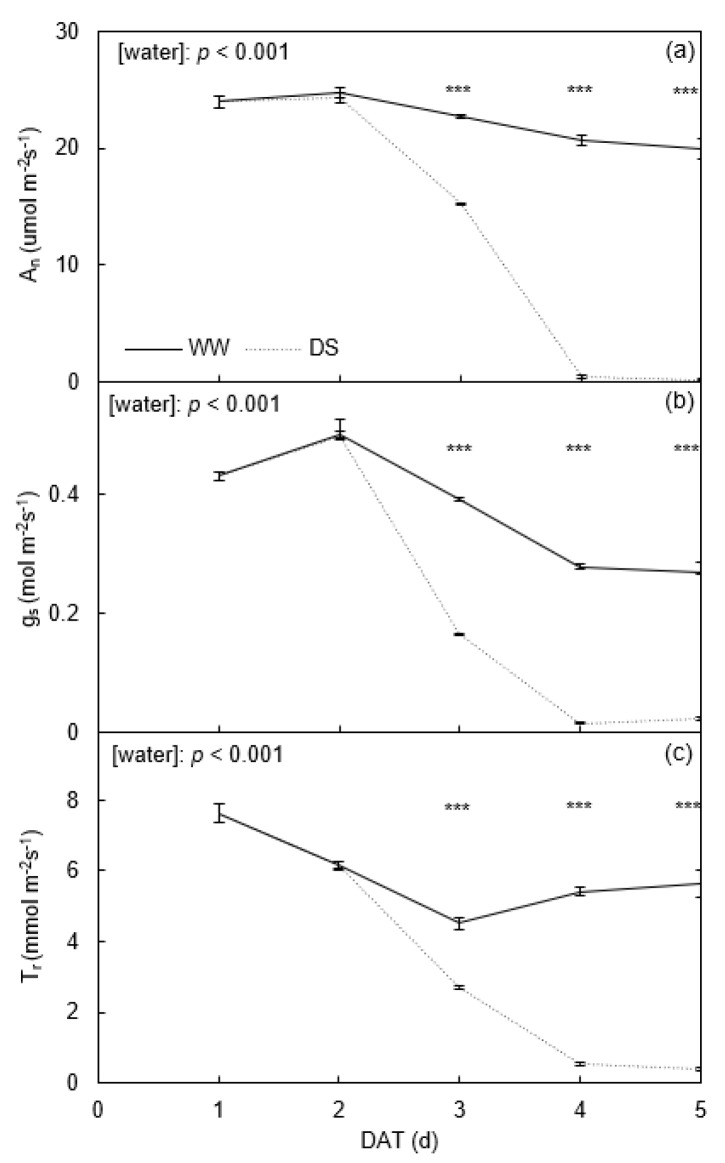
Changes in net photosynthetic rate (*A_n_*; (**a**)), stomatal conductance (*g_s_*; (**b**)) and transpiration rate (*T_r_*; (**c**)) of potato plants grown under well-watered (WW) and drought-stressed (DS) conditions in the field. Error bars indicate standard error of the mean (*n* = 5) and asterisks denote significant difference between treatments at *p* < 0.001. DAT denotes days after onset of treatment.

In the field experiment, *A_n_* of well-watered (WW) plants kept a high level of around 22.85 mol m^−2^ s^−1^ during the treatment period (Figure 2a). The *g_s_* of WW plants was between 0.27 to 0.50 mol m^−2^ s^−1^; similarly, *T_r_* of WW plants was between 4.9 to 7.6 mmol m^−2^ s^−1^, respectively (Figure 2b,c). After the onset of soil drying, the leaf gas exchange rates of drought stress (DS) plants remained similar to WW plants during the first 2 days; thereafter, they declined sharply and were significantly lower than WW plants (Figure 2).

**Figure 3:**
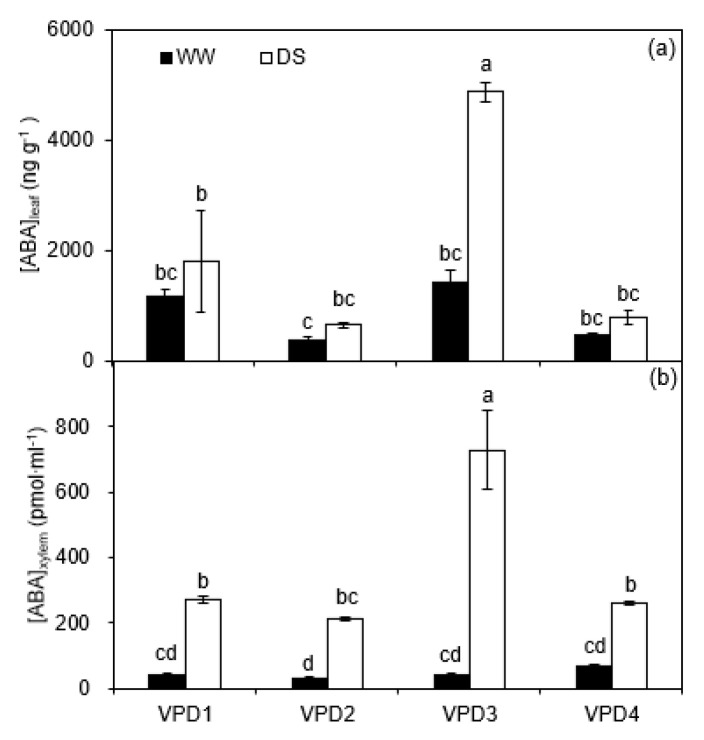
Concentrations of abscisic acid (ABA) in leaf (*[ABA]_leaf_*; (**a**)) and xylem (*[ABA]_xylem_*; (**b**)) of well-watered (WW) and drought-stressed (DS) potato plants grown under different vapor pressure deficits (VPD) in the greenhouse cells at final harvest. Error bars indicate the standard error of the mean (*n* = 3). Different small letters indicate significant difference at *p* < 0.05 level.

For the greenhouse experiment, the concentrations of ABA in leaf (*[ABA]_leaf_*) and xylem (*[ABA]_xylem_*) were significantly affected by temperature, relative humidity, and irrigation, as well as their combinations (Table 2). Across all treatments, DS plants had a higher *[ABA]_leaf_* than the WW plants, with a significant effect under normal temperature and low relative humidity (VPD3). Regardless of irrigation regimes, plants grown at normal temperature showed a significant difference in *[ABA]_leaf_* between high and low relative humidity under soil drying, in contrast to plants at high temperature with no differences (Figure 3a). The trends of ABA concentration in the xylem of WW and DS plants were similar to that in leaves (Figure 3b).

### 2.2. Leaf Water Potential, Stomatal Morphology, Leaf Area, and Specific Leaf Area

**Figure 4:**
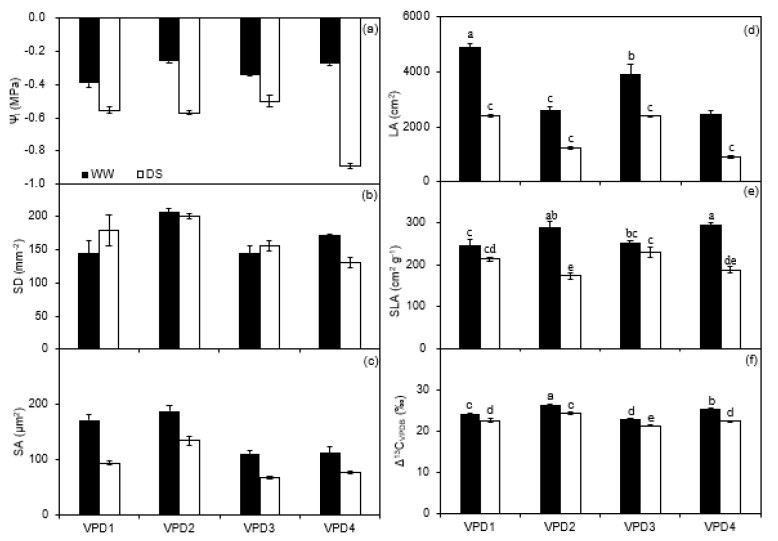
Leaf water potential (*ψ_l_*; (**a**)), stomatal density (*SD*; (**b**)), stomatal pore aperture (*SA*; (**c**)), leaf area (*LA*; (**d**)), specific leaf area (*SLA*; (**e**)), and leaf carbon isotope (*Δ^13^C*; (**f**)) of well-watered (WW) and drought-stressed (DS) potato plants grown under different vapor pressure deficits in the greenhouse cells at final harvest. Error bars indicate the standard error of the mean (*n* = 3). Plant water relations data throughout the growth period can be seen in Figure S2 in the Supplementary material. Different small letters indicate significant difference at *p* < 0.05 level.

In the greenhouse experiment, the leaf water potential (*ψ_l_*) of DS plants was lower than WW plants across all VPDs. The *ψ_l_* of DS plants under high temperature and low relative humidity (VPD4) was the lowest (−0.57 MPa) among the treatments (Figure 4a; Table 2). Plants grown at high temperatures had a higher stomatal density (*SD*) than those grown at normal temperatures (Figure 4b; Table 2). Moreover, WW plants had a higher stomatal pore aperture (*SA*) than DS plants at each VPD level. Regardless of irrigation, plants developed under high humidity had a higher SA as compared to those grown under low humidity at a given temperature (Figure 4c; Table 2).

Likewise, in the greenhouse, the leaf area (*LA*) of WW plants was higher than DS plants regardless of VPD regimes. Moreover, plants grown under low relative humidity had a lower *LA* as compared to those under high relative humidity at normal temperature; the *LA* of plants grown at normal temperature was higher than those grown at a high temperature (Figure 4d; Table 2). The specific leaf area (*SLA*) of WW plants was higher across all VPD levels as compared to the DS plants, especially at high temperatures: *SLA* of DS plants was 40 and 36% lower than WW plants under high and low relative humidity, respectively (Figure 4e; Table 2). *Δ^13^C* of WW plants was significantly higher than that of DS plants under each VPD level. Regardless of the irrigation treatment, plants developed at high temperatures had a higher *Δ^13^C* as compared to those grown at normal temperature under either relative humidity (Figure 4f; Table 2).

### 2.3. Shoot Dry Matter, Water Use, and Water Use Efficiency

**Figure 5:**
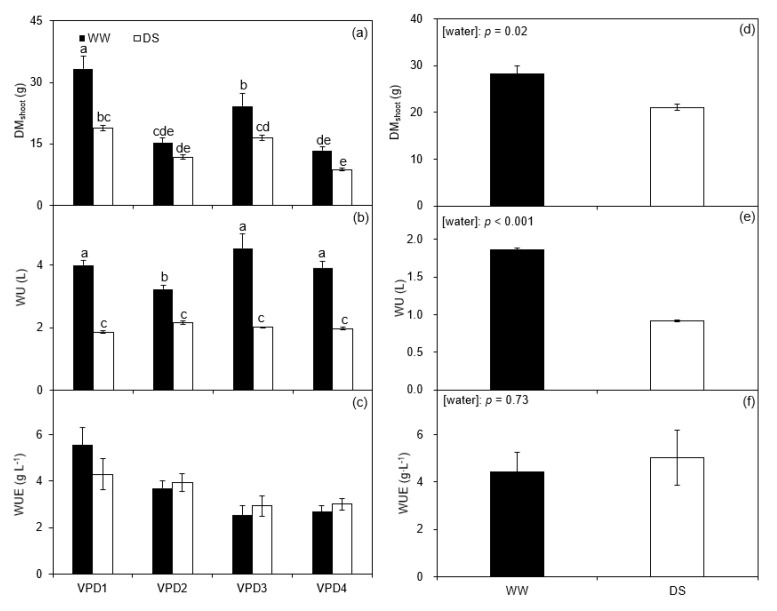
Shoot dry matter (*DM_shoot_*; (**a**,**d**)), water consumption (*WU*; (**b**,**e**)), and water use efficiency (*WUE*; (**c**,**f**)) of well-watered (WW) and drought-stressed (DS) potato plants grown under different vapor pressure deficits in the greenhouse cells and the field at final harvest. Error bars indicate the standard error of the mean (*n* = 3 or 5). Different small letters indicate significant difference at *p* < 0.05 level.

In the greenhouse, the dry matter of aboveground (*DM_shoot_*) of WW plants was higher than DS plants across all the VPD levels, especially under relative humidity at normal temperature. Moreover, the *DM_shoot_* of WW and DS plants developed at high temperature and low relative humidity (VPD4) was 34 and 25% higher than those grown at normal temperature and high relative humidity (VPD1; Figure 5a; Table 2). The water use (*WU*) of DS plants was lower than WW plants across all the VPD levels. Meanwhile, the *WU* of the WW plants grown at high temperature and relative humidity (VPD2) was lower than that for the other treatments (Figure 5b; Table 2), so was the *WUE* compared to the WW plants, though without apparent difference in *WUE* between the two irrigation treatments under each VPD treatment. In addition, the *WUE* of plants grown under low relative humidity was lower than that grown under high relative humidity at a given temperature; on the other hand, plants grown at high temperature had a lower *WUE* than those grown at normal temperature (Figure 5c; Table 2).

In the field experiment, *DM_shoot_* and *WU* of plants grown under WW conditions were 34 and 103% higher than DS, respectively (Figure 5d,e). DS plants had a 13.4% higher *WUE* as compared with WW plants, though the increase was not statistically significant (*p* = 0.73; Figure 5f).

### 2.4. Relationships between FTSW Threshold, g_s_, T_r_, WUE, and VPD

**Figure 6:**
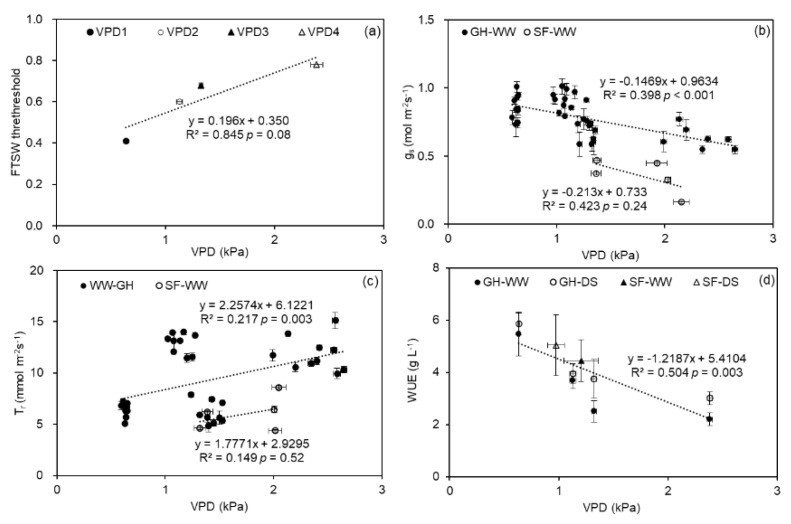
Relationship between vapor pressure deficit (VPD) and fraction of transpirable soil water (*FTSW*) threshold in the greenhouse cells (**a**), stomatal conductance (*g_s_*; (**b**)), and transpiration rate (*T_r_*; (**c**)) of well-watered (WW) potato plants grown in the greenhouse (GH) and field (SF), as well as their water use efficiency (*WUE*; (**d**)). The regression line is accompanied by an equation, significance level (*p*-value), and coefficient of determination (R^2^). Error bars indicate the standard error of the mean (*n*= 3 or 5).

In the greenhouse experiment, a positive linear relationship between VPD and the threshold of soil water content at which *T_r_* began to decrease was recorded (Figure 6a). For the WW plants in both experiments, *g_s_* and VPD were negatively correlated across all treatments (Figure 6b), while *T_r_* increased with the increasing VPD (Figure 6c). Finally, a significant linear decrease in *WUE* with increasing VPD was noticed (Figure 6d).

## 3. Discussion

Sole effects of high VPD or soil water deficits on plant growth and water relations have been intensively studied [5,27], while knowledge of their combined effects remains largely unstudied. In the future, more crops will be cultivated in arid environments attributing to the changing climate, whereas those in temperate environments become increasingly prone to multiple abiotic stresses such as prolonged and frequent droughts and heatwaves. Therefore, a deeper understanding of the underlying mechanisms for the response of plants to multiple stressors is required. This study provides empirical evidence for the responses of growth and physiological parameters of potato plants to drought and heat stress, individually or in combination.

### 3.1. Leaf Gas Exchange and Chemical Signaling

The gas exchange rates of potato leaves were sensitive to atmospheric and soil drought (Figure 1 and Figure 2; Table 1). Previous studies documented *A_n_* to decrease by stomatal and non-stomatal limitations under soil drying [28]. Here, the reduced *A_n_* of DS plants in the two experiments was mainly attributed to the reduced *g_s_*, related to the decreased intercellular CO_2_ concentration (Figure 1a,b), which corroborates previous results [11]. Shirke and Pathre (2004) also reported higher VPD (mainly caused by high temperature) to reduce the Rubisco activity, thereby reducing carboxylation efficiency and subsequently limiting carbon assimilation [29]. In the greenhouse experiment, the effect of high temperature or VPD on *A_n_* was insignificant before soil drying (Figure 1a; Table 1), which disagreed with previous studies [30] and likely relates to the relatively short period in the other studies compared to 5–7 weeks in the current investigation. Thus, the plants might have acclimated to such an environment and adjusted morphologically and photochemically. Consistent with the results of the greenhouse experiment, Wolf et al. (1990) reported that the photosynthetic system of potato plants has a high adaptability to high temperatures [31]. Nevertheless, in the field experiment, *A_n_* of the WW plants was significantly lower (*p* < 0.05) on the fourth day than on the third day, probably due to the increased VPD (Figure 1f and Figure 2a), corroborating previous findings [29].

**Figure 7:**
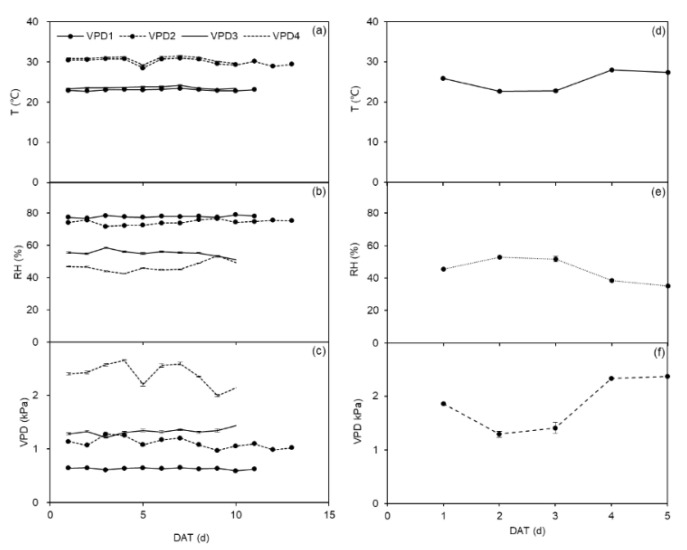
Mean daily temperature (T; (**a**,**d**)), relative humidity (RH; (**b**,**e**)), and vapor pressure deficit (VPD; (**c**,**f**)) in greenhouse cells and field conditions during the experimental period. Error bars indicate the standard error of the mean (*n* = 16). DAT denotes days after onset of treatment.

It was found in the greenhouse experiment that *g_s_* of potato plants decreased by soil drying accompanied by a higher *[ABA]_xylem_* and *[ABA]_leaf_* (Figure 1b and Figure 3). This result supports the consensus that the increased stomatal closure during progressive soil drying is induced by ABA signaling [32,33]. However, it should be noted that *g_s_* is controlled not only by soil water deficits but also atmospheric drought (e.g., high VPD), which could reduce *g_s_* even for WW plants [34,35]. In relation to the latter, it is interesting to point out the peculiar pattern found for the *g_s_* response to decreasing *FTSW*. The curves (Figure 1b; Table 1) fall in two distinct groups with significantly different thresholds of *g_s_* (C_g_), depending on relative humidity. However, VPD was fairly similar for three combinations of normal temperature and low relative humidity, compared to that at high temperature and high relative humidity (Figure 7c). The large difference in *C_g_* between the two treatments with similar VPD was likely due to their different levels of ABA concentration in both xylem sap and leaf (Figure 3). Thus, the significant differences in *C_g_* could not be ascribed to VPD alone, nor to *T_r_* (Figure 1c), but depended on temperature due to a differential ABA response to temperature. This funding has not been described before and should be considered when modelling plant transpiration. Under field conditions, fluctuations in temperature, relative humidity, and VPD are correlated, and *T_r_* is often modelled using the relations between *FTSW* and reference evapotranspiration (*ET_o_*) [36]. However, *ET_o_* aggregates a number of atmospheric variables and effects, thus, typically, it is not able to discriminate differences in FTSW thresholds between different temperatures at the same VPD level.

Plants grown under high relative humidity have a higher *g_s_* compared to those grown under low relative humidity due to modified stomata morphology, i.e., larger stomata length and pore aperture [37]. Similar results were also found in this study (Figure 1b and Figure 4c; Table 1 and Table 2). In addition, Merilo et al. (2018) found ABA was involved in mediating the response of *g_s_* to VPD [34]. In the present study, the results of the greenhouse experiment showed that *[ABA]_leaf_* of DS plants increased with increasing VPD under normal temperatures, suggesting ABA involvement in the regulations of gas exchange by VPD (Figure 3; Table S1). However, such an effect was not found under a high temperature, where both leaf and xylem ABA concentrations of WW and DS plants were not affected by relative humidity and were significantly lower than those grown under normal temperatures (Figure 3). The possible reasons behind this could be an inhibited inductive effect of drought stress on ABA concentration in potato plants due to high temperature as found in canola [38], or increased activity of ABA degrading enzymes at high temperature as found in rose [39], thereby resulting in a lower ABA concentration.

Stomata’s response to relative humidity relies on sensing *T_r_* rather than relative humidity [40]. In both experiments, soil water deficits significantly limited *T_r_*, which was due to the decrease in *g_s_* during the experimental periods (Figure 1b,c and Figure 2b,c; Table 1), the correspondingly lowered *A_n_* also led to a lower shoot dry matter (Figure 1a and Figure 5a,d). In the greenhouse experiment, *T_r_* of potato plants increased with the increasing temperature under either relative humidity (Figure 1c; Table 1); likewise, in the field experiment, *T_r_* of potato plants was significantly greater on the fourth day due to the increased VPD, which resulted from the high temperature and low relative humidity (Figure 2c and Figure 7d,f). Montero et al. (2001) also reported elevated temperature or VPD to trigger an increased atmospheric evaporative demand and result in a higher *T_r_* [41]. In addition, these results indicate that plants grown under high temperatures might have a higher leaf cooling capacity resulting from the increase in *T_r_* as compared with those grown under low temperatures [42]. Furthermore, the high temperature would limit the growth of plants and lead to a lower yield and *WUE* [43]. Zhang et al. (2020) found that *T_r_* of plants developed under high relative humidity was higher than in those grown under low relative humidity at a given temperature, which was in line with the results here [26]. A plausible explanation is that the malfunctioning stomata caused by high relative humidity weaken the capacity of the plant to control water loss. The results of the greenhouse experiment showed that *T_r_* of plants developed under high temperature and relative humidity was significantly higher than other treatments before imposing soil drying (Figure 1c; Table 1), which was caused by a higher evaporation demand at high temperature and compromised stomatal closure (caused by high relative humidity). Therefore, plants grown in a hot and wet environment would be stressed more when soil water deficits and atmospheric drought occur simultaneously [32].

In the greenhouse experiment, *FTSW* was fitted in a linear plateau model to evaluate the response of leaf gas exchange rates to progressive soil drying [44]. The results of Ray and Sinclair (1998) for maize (*Zea mays* L.) and soybean (*Glycine max* L.) also pointed to soil water content as the key factor determining the response of transpiration to soil water deficit, regardless of the size of plants or pots [27]. Here, after the onset of soil drying, there was no notable difference in the *FTSW* threshold for *A_n_* (*C_A_*) between the four VPDs (Figure 1a; Table 1), indicating soil water deficit to likely have dominated over the effects of VPD on *A_n_*. However, the *FTSW* threshold for *g_s_* (*C_g_*) and *T_r_* (*C_T_*) increased with increasing VPD at a given temperature. Grossiord et al. (2020) suggested that the similar sensitivity of *g_s_* and *T_r_* to soil drying under different VPD levels was due to the decreased *T_r_* as the soil desiccates, mainly due to decreased *g_s_* [27]. Furthermore, the results suggest that *g_s_* and *T_r_* of potato plants became more sensitive to soil drying under high VPD (Figure 1b,c; Table 1). Numerous studies have focused on the response of plant transpiration to soil drying and documented that *T_r_* would not decline until the fraction of available water remaining in the soil had reached 0.3 to 0.4 [45]. This study clearly shows that the *C_T_* of potato plants during soil drying under different VPDs ranged from 0.41 to 0.78, which was higher than previously reported for other plants (e.g., peanut, 0.22–0.71) [46]. These results suggest that potato plants are more sensitive to soil drying than other crop species and could conserve water by limiting maximum *T_r_* under high VPD. In addition, the low *T_r max_* and *C_T_* under low VPD (viz., normal temperature and high relative humidity) were consistent with results reported by Sinclair and Ludlow (1986), indicating that low evaporative demand could ensure continuous water flow in the soil–plant–atmosphere continuum to achieve an unlimited *T_r_* until water availability in the soil decreased to a relatively low level [47]. The same difference in *C_T_*, but less pronounced than for *C_g_*, was seen between normal temperature and low relative humidity and high temperature and high relative humidity treatments with similar VPD.

### 3.2. Leaf Water Relations, Stomatal Morphology, Leaf Area, and Specific Leaf Area

A previous study with potatoes reported a negative relationship between *g_s_* and ABA in DS plants, which was not evident in the current study when examining the data under high-temperature treatment in the greenhouse, likely due to high temperature promoting ABA degradation in the leaf, or increased ABA dilution in the xylem [43]. On the other hand, the high temperature decreased *ψ_l_* under soil drying, especially combined with low relative humidity conditions (VPD4; Figure 4a; Table 2 and Tables S1–S3) and attributed to the increased *T_r_* [48]. In addition, it has been reported that plants grown under high relative humidity show higher *ψ_l_* due to the low transpirational water dissipation, in line with the results detected here in the WW plants in the greenhouse experiment (Figure 4a; Table 2). Noteworthy is that with increasing intensity of stress, the sap movement, and consequently the velocity of the ABA transport, is reduced, whereas the hydraulic signal is strengthened. To this end, Marino et al. (2017) showed that under water deficit conditions, the hydraulic signal sustained the leaf ABA biosynthesis by keeping the chloroplastic 2-C-methylerythritol-5-phosphate (MEP) pathway active [49]. Future studies could investigate whether and to what extent the MEP pathway increased in the potato roots under increasing water stress.

Genetic and environmental differences are the main cause of differences in *SD* [50]. The SD of the potato plants increased with increasing temperature in the greenhouse experiment (Figure 4b; Table 2), as was also found for soybean [51]. The leaf is the main plant organ sensitive to the environment and *LA* was significantly reduced by soil water deficits and high VPD (caused by high T; Figure 4d; Table 2 and Table S1), which agrees with Yang et al. (2012), indicating high VPD or soil drying to reduce dry matter accumulation by limiting *LA* [7]. This could be due to three reasons: (1) a decrease in leaf cell expansion attributed to the decreased turgor pressure by soil drying; (2) high temperature could accelerate protein degradation, thus causing senescence and death; (3) elevated VPD reduced *LA* through modifying cell number (i.e., epidermal cell division) [52,53,54]. On the other hand, Stuerz and Asch (2019) reported that the leaf expansion rate of plants developed under high relative humidity was higher than that under low relative humidity, which was also confirmed in this study [55]. For WW plants in the greenhouse experiment, *LA* increased with the increasing relative humidity at a given temperature.

The *SLA* of the potato plants in the greenhouse experiment decreased under soil water deficits (significantly at high temperature; Figure 4e; Table 2), indicating that plants with low *SLA* (thicker leaves) could assimilate more CO_2_ due to the high nitrogen content and more mesophyll cells per unit area, thus, leading to a higher yield and *WUE* [56]. In addition, the high temperature increased *SLA* in WW plants was also an adaptive pattern to avoid heat damage, as heat dissipation capacity would be higher in thinner than thicker leaves [57]. In the greenhouse experiment, the DS plants grown at high temperatures had lower *LA* and *SLA* than those under normal temperatures, indicating that soil drying at a high temperature has negatively affected plant physiology and growth to a larger extent compared to normal temperatures. The *Δ^13^C* is considered an indicator of plant *WUE* and the lower values for DS plants in the present study (Figure 4f and Figure S1), as was also found by Beerling and Woodward (1995), indicating a negative correlation between *Δ^13^C* and *WUE* [58].

### 3.3. Shoot Dry Matter, Water Use, and Water Use Efficiency

The growth of potato plants was significantly constrained by soil water deficits in both the greenhouse and the field experiments (Figure 5a,d; Table 2), as also reported for tomato, maize, and soybean [59]. In the greenhouse experiment, soil drying exacerbated the inhibition of potato growth under high VPD at a given temperature, consistent with the results of Rashid et al. (2018) [60]. Although *T_r_* increased at high VPD (i.e., hot-wet environment), it could not offset the negative effect of reduced *LA* on plant water consumption under such conditions (Figure 1c, Figure 4c and Figure 5b) [37]. Crop *WUE* could be improved through partial stomatal closure and reducing *Tr* under moderate soil water deficits, and *WUE* of potato plants grown under soil water deficits was improved in the greenhouse and field experiments (Figure 5c,f).

### 3.4. Relationships between FTSW Threshold, g_s_, T_r_, WUE, and VPD

Cunningham (2004) found that stomata and transpiration were responsive to VPD, as also observed here in both experiments by reduced *g_s_* of WW plants with increasing VPD, while *T_r_* increased (Figure 6b,c) [61]. In addition, there was a common negative correlation between *WUE* and VPD across the data in both experiments (Figure 6d; Table S1). Such a relationship could be explained by the increased atmospheric evaporative demand by VPD and the resulting higher *T_r_*, which increased plant water loss while limiting growth, particularly under soil drying circumstances. Nevertheless, the relative contributions of temperature and relative humidity to *WUE* remain unclear (Table 2) and merit further investigation.

## 4. Materials and Methods

### 4.1. Greenhouse and Field Experiments

The greenhouse experiment was conducted at the Faculty of Science, Copenhagen University, Taastrup, Denmark. Tubers of *Solanum tuberosum* L. cv. *Oleva* (40–55 mm in diameter) were planted in a depth of 3 cm in 2.5 kg peat substrate (Krukväxtjord, Sweden; organic matter >95%, pH = 5.5–6.5, EC = 2.5–3.5 ms cm^−1^) filled into 108 cylindrical pots (4 L, 15 cm diameter and 23 cm height). Fertilizer was applied to each pot with 1 g N (NH_4_NO_3_), 0.8 g P, and 1 g K (H_2_KPO_4_). Prior to planting, potato tubers were pre-sprouted for two weeks at 12–14 °C with dim overhead light. Only one sprout was left to emerge during the germination phase. The tubers were planted in four greenhouse cells, and the VPD treatments in each cell are shown in Table 3. The photoperiod was 16 h with above 500 μmol m^−2^ s^−1^ photosynthetic active radiation provided by sunlight plus LED lamps in each greenhouse cell. Figure 7a–c shows the dynamic change of temperature, relative humidity, and VPD in the four greenhouse cells during the greenhouse experimental periods.

The field experiment followed the greenhouse experiment and was carried out from May to June 2019 at the experiment station of Copenhagen University, Taastrup, Denmark. On 21 May, two potato tubers were sown in 25 pots (24 L) containing 21.5 kg of air-dried sandy soil with a bulk density of 1.44 g cm^−3^. The soil had a volumetric soil water content of 21 and 5% at field capacity (FC) and permanent wilting point, respectively. Each pot was fertilized with three granular compound (NPK) fertilizers: 2.1 g N, 0.5 g P, and 2 g K. The treatment before planting and at the germination of the potato tubers was analogous to that in the greenhouse experiment. All pots were placed under a rainout shelter to prevent rainfall. Figure 7d–f shows the dynamic change of temperature, relative humidity, and VPD during the field experimental periods.

### 4.2. Irrigation Treatments

In all pots, soil water status was measured daily by either weighing the pots in the greenhouse experiment or monitoring by time-domain reflectometer (TDR, Soil Moisture Equipment Crop., Santa Barbara, USA) with 20 cm probes installed in the middle of each pot in the field experiment, read at 1500 hours and expressed as a fraction of transpirable soil water (*FTSW*) [62].

In the greenhouse experiment, the potato plants in all pots were irrigated daily to 95% of pot water holding capacity before the start of the drought treatment. Three weeks after emergence, a 2-cm perlite layer was covered on the soil surface to minimize evaporation, and three plants from each cell (VPD treatment) were randomly selected and harvested to determine the initial growth and physiological parameters before the onset of the drought treatment. Afterwards, in each greenhouse cell, 12 plants were kept well-irrigated (WW; control), and the other 12 pots were drought-stressed (DS) by withholding irrigation until the daily transpiration rate decreased to ca. 10% of the WW pots. During the progressive soil drying treatment, the plants were harvested four times at the daily transpiration rate of DS plants decreased to 70, 50, 30, and 10% of the WW plants, respectively (three plants per treatment in each greenhouse cell). In addition, the soil water content data were subjected to daily normalizations following the method mentioned by [63], while the leaf gas exchange data were not normalized due to the environment in the greenhouse cells being well controlled.

In the field experiment, irrigation treatment was commenced four weeks after planting, and five pots were randomly harvested for initial data collection before progressive soil drying. Afterwards, 10 pots were well-watered, while no irrigation was applied to the remaining 10 pots until their evapotranspiration was 10% of the WW plants. During the irrigation treatment period, five plants per treatment were randomly harvested when the daily evapotranspiration of DS plants decreased to 50 and 10% of the WW plants, respectively.

### 4.3. Variables Evalated

**Figure 8:**
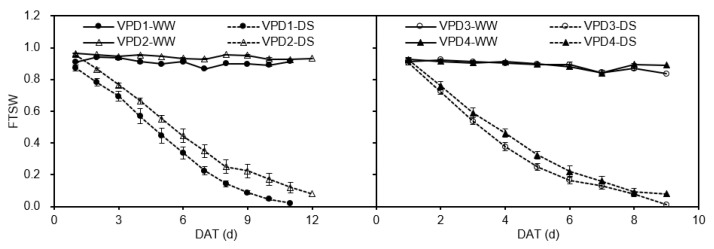
Trends of fraction of transpiration soil water (*FTSW*) at days after imposing treatment stress (DAT) for well-watered (WW) and drought-stressed (DS) potato plants grown under different vapor pressure deficits (VPD) in the greenhouse cells. Error bars indicate the standard error of the mean (*n* = 3). DAT denotes days after onset of treatment.

The total transpirable soil water (*TTSW*) was calculated for all pots as the difference between pot weights at 100% WW holding capacity (i.e., 4.9 and 24.3 kg in the greenhouse and field experiments, respectively) and when transpiration and evapotranspiration of the DS plants decreased to 10% of the control WW plants (i.e., 2.1 and 22.5 kg in the greenhouse and field experiments, respectively). The daily value of *FTSW* was estimated as the ratio between the amount of transpirable soil water that was remaining in the pots and *TTSW* [53]:

*FTSW = (WT_n_ − WT_f_)/TTSW* (1)

where *WT_n_* (g) is pot weight on a given date, *WT_f_* (g) is pot weight when daily transpiration of the stressed plants was 10% of the well-watered plants. Figure 8 shows the dynamic changes of *FTSW* during the whole experiment period in each greenhouse cell.

### 4.4. Leaf Gas Exchange and Chemical Signals

After the onset of the DS treatment, leaf gas exchange rates, including *A_n_* (μmol m^−2^ s^−1^), *g_s_* (mol m^−2^ s^−1^), and *T_r_* (mmol m^−2^ s^−1^), were measured daily on the latest fully expanded leaf (one leaf per plant, three plants in the greenhouse, and five plants in the field, both per treatment) from 0930 to 1230 h at 1500 μmol m^−2^ s^−1^ photosynthetic light saturation and 400 ppm CO_2_ with a LiCor-6400XT portable photosynthetic system (LI-COR, Inc., Lincoln, NE, USA). Prior to the measurements, the equipment was supplied with a new desiccant and preheated for 30 min, followed by calibration of both H_2_O and CO_2_. During the measurements, the leaf chamber environment (i.e., temperature, relative humidity, and CO_2_) were set according to the conditions in the respective greenhouse cells or the field.

The concentrations of ABA in leaf and xylem were determined only in the greenhouse experiment. After leaf gas exchange measurement, three leaves (one leaf per plant) per treatment were cut and immediately packed in aluminum foils and frozen in liquid nitrogen, then stored at −80 °C for the measurement of leaf ABA concentration (*[ABA]_leaf_*). Xylem sap was collected by pressurizing the roots of the potted plants in a pressure chamber. The whole pot was inserted into the pressure chamber and sealed, leaving only the shoot, which was then cut at 3–5 cm height from the stem base, and the cut surface was dried with blotting paper. Pressure was applied slowly until the xylem sap outflowed from the cut. Pipettes were used to collect the xylem sap (0.5 mL) from the cutting surface into an Eppendorf vial wrapped with aluminum. All sap samples were frozen immediately and stored at −80 °C for ABA analysis.

Leaf samples were put into a mortar and added to the liquid nitrogen, then immediately ground to powder measuring 27–33 mg per sample in a 1.5 mL centrifuge tube. All the samples were extracted with 1.0 mL milli-Q water on a shaker for 16 h in a 4 °C environment after weighing. The samples were centrifuged at 14,000 r/min for 5 min in a 4 °C environment, and supernatant was added of 0.7 mL to a 1.5 mL centrifuge tube for *[ABA]_leaf_* analysis. *[ABA]_leaf_* and ABA concentration in xylem sap (*[ABA]_xylem_*) were determined by enzyme-linked immunosorbent assay (ELISA) [64], which is a less sensitive but fast and affordable method with high enough detection limits for studying plant-level responses involving a wide range of drought treatments, compared to gas or liquid chromatography and coupled mass spectrometry, which contain complicated operation, high costs, and require tedious purification steps prior to the chromatographic separation in order to remove background contaminants in the sample [65].

### 4.5. Leaf Water Potential, Stomatal Morphology, Leaf Area, and Specific Leaf Area

In the greenhouse, leaf water potential (*ψ_l_*) was measured on the fully expanded leaves after measuring gas exchange at final harvest using a pressure chamber (Soil Moisture Equipment, Santa Barbara, CA, USA). Three plants per treatment were selected to collect epidermal imprint of leaf after leaf gas exchange measurement. Leaves were wipe-cleaned with degreased cotton and daubed with nail polish to the middle area between the edge and the central vein and left for 30 min [18]. The epidermal impressions were peeled off from the leaves with transparent tape and observed under a photomicroscope system (Leica, D 35530, Wetzlar, Germany) equipped with a digital camera, with images analyzed by image-editing software (Leica, ver. 2.5.0, CMS GmbH Switzerland). Stomata number was counted on the images (nine images per treatment), and stomatal pore aperture (*SA*, μm^2^) was calculated (three replicates per treatment; 6 stomata for one replication) as [18]:

*SA* = (π × *S_l_* × *S_w_*)/4 (2)

where *S_l_* is stomatal pore aperture length and *S_w_* is stomatal pore aperture width. These two parameters were also measured by image-editing software.

A portable leaf area meter (Li-3100, Li-Cor Inc., Lincoln, NE, USA) was used to collect the leaf area (*LA*, cm^2^) parameters. Moreover, the specific leaf area (*SLA*, cm^2^ g^−1^) was calculated as:

*SLA = LA/DM_leaf_* (3)

where *DM_leaf_* is the leaf dry matter.

At the last harvest, leaf samples used for carbon isotope discrimination (*Δ^13^C*) determination were dried in the oven at 70 °C until constant weight, then the dried samples were ground to a fine powder for *Δ^13^C* analysis, and the measurements were performed at the University of California Davis Stable Isotope Facility in the USA.

### 4.6. Shoot Dry Matter, Water Use, and Water Use Efficiency

For all pots in the two experiments, shoot dry matter (*DM_shoot_*, g) was determined after oven-drying the harvest samples at 70 °C until constant weight. Plant water consumption (*WU*, L) was calculated as the total irrigation water used during the whole treatment period plus the changes in the soil water content. Plant *WUE* (g L^−1^) was calculated as:

*WUE = ΔDM/WU* (4)

where *ΔDM* is the increment of *DM_shoot_* during the treatment period.

### 4.7. Data Analyses and Statistics

For the greenhouse experiment data, the linear-plateau model was used to describe the responses of leaf gas exchange rates to *FTSW* [66]:

if *FTSW* > *C_i_*, *y* = *y_max_* (5)

if *FTSW ≤ C_i_, y = y_max_ + α ×* (*FTSW−C_i_*) (6)

where *y* is either *A_n_*, *g_s,_* or *T_r_*, or *y_max_* is either *A_n max_*, *g_s max_,* or *T_r max_*, *C_i_* is the threshold of *FTSW* at which the measured leaf gas exchange rates started to decrease, and *α* is the slope of the linear Equation. In addition, the PROC NLIN of SAS (ver. 9.2; SAS Institute Inc., Cary, NC, USA) was used to estimate *y_max_*, *α*, and *C_i_* in the model.

Two- and three-way ANOVA were used to examine the effects of main factors and their interactions on physiological parameters. The differences between treatments were compared by the Duncan test. The performances of relationships between the measured parameters were determined by the regression analysis.

## 5. Conclusions

In conclusion, soil water deficits significantly limited leaf gas exchange rates, *ψ_l_*, *SA*, *LA*, and *SLA*, contributing to reduced *DM_shoot_*, while increasing *[ABA]_leaf_* and *[ABA]_xylem_* at each VPD level. High VPD significantly affected *g_s_*, *T_r_*, plant ABA concentration, *ψ_l_*, *SA*, *SD*, *LA*, *DM_shoot_,* and *WUE*, while *g_s_* and *WUE* were negatively correlated with VPD and *T_r_* was positively correlated with VPD. Furthermore, the results demonstrated that the plant water relation and dry matter of potatoes developed well under low VPD, while the combined effects of high VPD and soil water deficits had significantly on *[ABA]_leaf_*, *[ABA]_xylem_*_,_ *LA* and *SLA* exacerbated the adverse effects on plant growth and development. With respect to *g_s_*, the *FTSW* threshold depended on temperature due to a differential ABA response to temperature, apparently affecting the ABA concentration. This is a novel and important finding for ecosystem studies and modelling actual *T_r_*. In summary, the results provide essential and detailed insights into plant mechanisms of stomatal control and water status under varied VPD and soil moisture conditions, which are essential for developing mechanistic models predicting crop *WUE* in a future arid climate.

## Figures and Tables

**Table 1:** Results of the linear-plateau regression of leaf net photosynthesis rate (*A_n_*), stomatal conductance (*g_s_*) and transpiration rate (*T_r_*) to the reduction in fraction of transpirable soil water (*FTSW*) for the potato plants grown in the greenhouse cells. Data are shown in Figure 1.

Treatment	*A_n_* *A_n max_* (μmol m^−2^ s^−1^)	*C_A_*	*g_s_* *g_s max_* (mol m^−2^ s^−1^)	*C_g_*	*T_r_* *T_r max_* (mmol m^−2^ s^−1^)	*C_T_*
VPD1	22.23 (21.04–23.42)	0.40 (0.35–0.44)	0.81 (0.75–0.87)	0.43 (0.37–0.50)	4.96 (4.57–5.35)	0.41 (0.35–0.47)
VPD2	24.94 (23.21–26.66)	0.38 (0.28–0.49)	0.88 (0.81–0.95)	0.47 (0.38–0.55)	10.69 (9.93–11.46)	0.60 (0.46–0.75)
VPD3	22.38 (20.78–23.98)	0.47 (0.39–0.56)	0.77 (0.70–0.85)	0.74 (0.64–0.84)	9.35 (8.63–10.06)	0.68 (0.60–0.77)
VPD4	21.91 (19.97–23.86)	0.53 (0.43–0.62)	0.60 (0.52–0.69)	0.80 (0.66–0.94)	14.99 (13.16–16.81)	0.78 (0.64–0.91)

Note: *A_n max_*, *g_s max_*, and *T_r max_* indicated the initial values of the parameters when the plants were not significantly affected by drought; *C* (*C_A_*, *C_g_,* or *C_T_*) indicated the threshold at which the parameter (*A_n_*, *g_s_*, or *T_r_*, respectively) start to decrease due to drought stress. Values in the parentheses are 95% confidence intervals of the parameters.

**Table 2:** Results of a three-way analysis of variance for abscisic acid concentration (ABA) in leaf (*[ABA]_leaf_*) and xylem (*[ABA]_xylem_*), leaf water potential (*ψ_l_*), stomatal density (*SD*), stomatal pore aperture (*SA*), leaf area (*LA*), special leaf area (*SLA*), leaf carbon isotope (*Δ^13^C*), shoot dry matter (*DM_shoot_*), water consumption (*WU*), and water use efficiency (*WUE*) of potato plants as affected by air temperature (T, 23 and 30 °C), air relative humidity (RH, 50 and 75%) and irrigation (Irr, either full or nil) in greenhouse cells at final harvest. Data are shown in Figure 1, Figure 2, Figure 3, Figure 4 and Figure 5.

Factor	[*ABA*]*_leaf_* (ng g^−1^)	[*ABA*]*_xylem_* (pmol ml^−1^)	*Ψ_l_* (MPa)	*SD* (mm^−2^)	*SA* (μm^2^)	*LA* (cm^2^)	*SLA* (cm^2^ g^−1^)	*Δ^13^C* (‰)	*DM_shoot_* (g)	*WU* (L)	*WUE* (g L^−1^)
[T]	***	**	*	**	*	***	ns	***	***	ns	*
[RH]	**	**	*	ns	***	*	ns	***	*	ns	***
[Irr]	**	***	***	ns	***	***	***	***	***	***	ns
[T*RH]	*	*	ns	ns	ns	ns	ns	ns	ns	ns	ns
[T*Irr]	*	**	ns	ns	ns	ns	***	*	*	**	ns
[RH*Irr]	*	**	ns	ns	*	ns	ns	ns	ns	ns	ns
[T*RH*Irr]	*	**	ns	ns	ns	ns	ns	ns	ns	ns	ns

Note: *, **, and *** indicate significance levels at *p* < 0.05, *p* < 0.01, and *p* < 0.001, respectively; ns denotes no significant.

**Table 3:** Set points for VPD treatments in greenhouse cells.

Greenhouse Cells	VPD Treatments	T (°C)	RH (%)	VPD (kPa)	CO_2_ (ppm)
I	VPD1	23	75	0.70	400
II	VPD2	30	75	1.06	400
III	VPD3	23	50	1.40	400
IV	VPD4	30	50	2.12	400

T, temperature; RH, relative humidity; VPD, vapor pressure deficit.

## Data Availability

Not applicable.

## Supplementary Materials

The following supporting information can be downloaded at: https://www.mdpi.com/article/10.3390/plants11091126/s1, Figure S1: Relationship between leaf carbon isotope discrimination (*Δ^13^C*) and water use efficiency (*WUE*) of well-watered (WW) and drought-stressed (DS) potato plants grown in the greenhouse cells at final harvest. The regression line is accompanied by an equation, significance level (*p*-value), and coefficient of determination (*R*^2^); Figure S2: Leaf water potential (*ψ_l_*), leaf area (*LA*), and special leaf area (*SLA*) of well-watered (WW; a, c, e) and drought-stressed (DS; b, d, f) potato plants grown under different vapor pressure deficit in the greenhouse cells at different harvest times. Error bars indicate the S.E. (*n* = 3). Values for WW and DS plants are equal at first harvest, after which irrigation treatment commenced. VPD1, normal temperature and high relative humidity; VPD2, high temperature and relative humidity; VPD3, normal temperature and low relative humidity; VPD4, high temperature and low relative humidity. Table S1: Two-way analysis of variance for abscisic acid concentration in leaf (*[ABA]_leaf_*) and xylem (*[ABA]_xylem_*), leaf water potential (*ψ_l_*), stomatal density (*SD*), stomatal pore aperture (*SA*), leaf area (*LA*), special leaf area (*SLA*), leaf carbon isotope (*Δ^13^C*), shoot dry matter (*DM_shoot_*), water consumption (*WU*), and water use efficiency (*WUE*) of potato plants grown under different vapor pressure deficits (VPD1, normal temperature and high relative humidity; VPD2, high temperature and relative humidity; VPD3, normal temperature and low relative humidity; VPD4, high temperature and low relative humidity) and irrigation (Irr, full or nil) at the final harvest. Data are shown in Figure 3, Figure 4 and Figure 5; Table S2: Three-way analysis of variance for leaf water potential (*ψ_l_*), leaf area (*LA*), and special leaf area (*SLA*) of potato plants grown under different vapor pressure deficits (VPD1, normal temperature and high relative humidity; VPD2, high temperature and relative humidity; VPD3, normal temperature and low relative humidity; VPD4, high temperature and low relative humidity) and irrigation (Irr, full or nil) at different harvest times; Table S3: Two-way analysis of variance for leaf water potential (*ψ_l_*), leaf area (*LA*), and special leaf area (*SLA*) of potato plants grown under different vapor pressure deficit (VPD1, normal temperature and high relative humidity; VPD2, high temperature and relative humidity; VPD3, normal temperature and low relative humidity; VPD4, high temperature and low relative humidity) and irrigation (Irr, full or nil) at different harvest times.
