# Supplementary material for: Physiological and Growth Responses of Potato (Solanum Tuberosum L.) to Air Temperature and Relative Humidity under Soil Water Deficits
Source: Plants (Basel). 2022 Apr 21;11(9):1126. doi: 10.3390/plants11091126 (PMC9105088; doi:10.3390/plants11091126)
Supplement: Supplementary file 1 [file plants-11-01126-s001.zip › plants-1667529-supplementary.pdf]

plants

Physiological and growth responses of potato (*Solanum tuberosum* L.) to air temperature and relative humidity under soil water deficits

Peng Zhang[1,2,4,5], Xin Yang[2,3,4], Kiril Manevski[5,6], Shenglan Li[2], Zhenhua Wei[4], Mathias Neumann Andersen[5,6], Fulai Liu[2,6*]

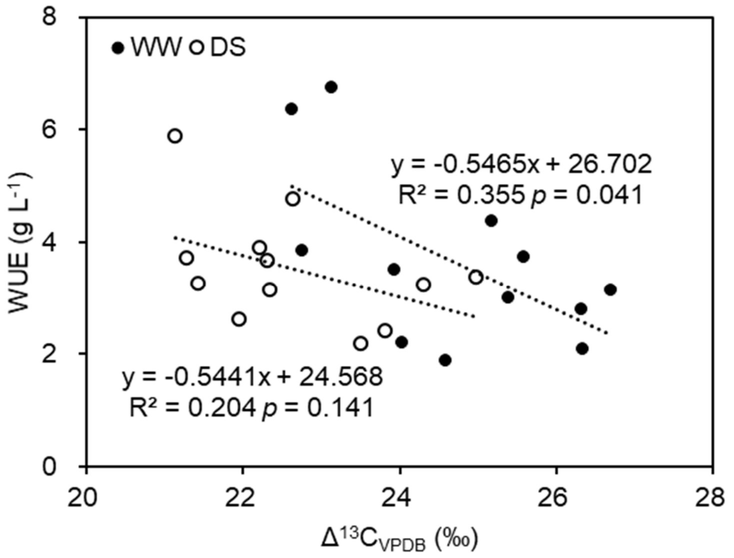

Figure S1. Relationship between leaf carbon isotope discrimination ($\Delta\ ^{13}C$) and water use efficiency (*WUE*) of well-watered (WW) and drought-stressed (DS) potato plants grown in the greenhouse cells at final harvest. Regression line is accompanied with equation, significance level (*p*-value) and coefficient of determination (R^2).

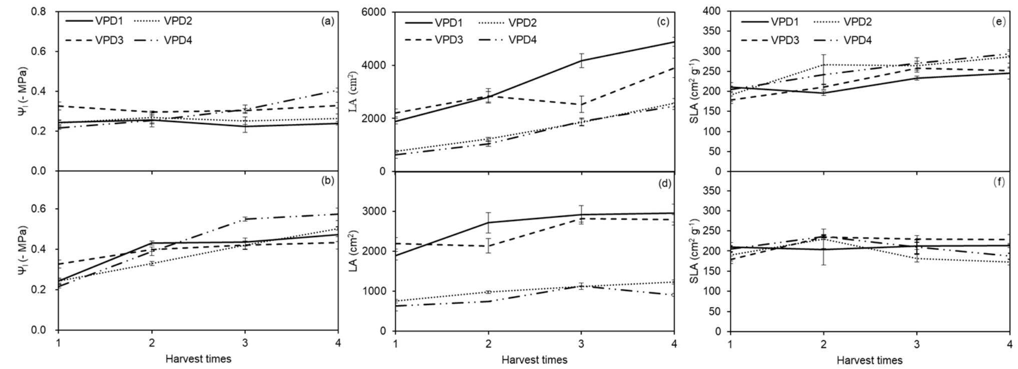

Figure S2. Leaf water potential (ψ_l), leaf area (*LA*) and special leaf area (*SLA*) of well-watered (WW; a, c, e) and drought-stressed (DS; b, d, f) potato plants grown under different vapor pressure deficit in the greenhouse cells at different harvest times. Error bars indicate the S.E. (n = 3). Values for WW and DS plants are equal at first harvest, after which irrigation treatment commenced. VPD1, normal temperature and high relative humidity; VPD2, high temperature and relative humidity; VPD3, normal temperature and low relative humidity; VPD4, high temperature and low relative humidity.

Table S1. Two-way analysis of variance for abscisic acid concentration in leaf (*$[ABA]_{leaf}$*) and xylem (*$[ABA]_{xylem}$*), leaf water potential (ψ_l), stomatal density (*SD*), stomatal pore aperture (*SA*), leaf area (*LA*), special leaf area (*SLA*), leaf carbon isotope ($\Delta^{13}C$), shoot dry matter (*DM_{shoot}*), water consumption (*WU*) and water use efficiency (*WUE*) of potato plants grown under different vapor pressure deficit (VPD1, normal temperature and high relative humidity; VPD2, high temperature and relative humidity; VPD3, normal temperature and low relative humidity; VPD4, high temperature and low relative humidity) and irrigation (Irr, full or nill) at the final harvest. Data are showed in Figures. 3-5.

Factor	$[ABA]_{leaf}$ (ng g^{-1})	$[ABA]_{xylem}$ (pmol ml^{-1})	Ψ_l (MPa)	SD (mm^{-2})	SA (μm^2)	LA (cm^2)	SLA (cm^2 g^{-1})	$\Delta^{13}C$ (‰)	DM_{shoot} (g)	WU (L)	WUE (g L^{-1})
[Irr]	**	***	***	ns	***	***	***	***	***	***	ns
[VPD]	***	**	**	*	***	***	ns	***	***	ns	**
[Irr]×[VPD]	**	**	ns	ns	ns	*	**	*	*	*	ns

Note: *, ** and *** indicate significance levels at $p < 0.05$, $p < 0.01$ and $p < 0.001$, respectively; ns denotes no significant.

Table S2. Three-way analysis of variance for leaf water potential (ψ_l), leaf area (*LA*) and special leaf area (*SLA*) of potato plants grown under different vapor pressure deficit (VPD1, normal temperature and high relative humidity; VPD2, high temperature and relative humidity; VPD3, normal temperature and low relative humidity; VPD4, high temperature and low relative humidity) and irrigation (Irr, full or nill) at different harvest times.

Factor	Ψ_l (MPa)				LA (cm^2)				SLA ($cm^2\ g^{-1}$)			
	Harvest times				Harvest times				Harvest times			
	1	2	3	4	1	2	3	4	1	2	3	4
[T]	**	**	*	*	***	***	***	***	ns	ns	ns	ns
[RH]	*	*	ns	**	ns	ns	ns	ns	ns	ns	ns	ns
[Irr]	**	***	***	***	*	ns	*	*	*	*	*	***
[T×RH]	**	*	ns	*	ns	ns	ns	ns	ns	ns	ns	ns
[T×Irr]	*	ns	ns	ns	ns	ns	ns	ns	ns	ns	ns	*
[RH×Irr]	ns	ns	ns	ns	ns	ns	ns	ns	ns	ns	ns	ns
[T×RH×Irr]	ns	ns	ns	ns	ns	ns	ns	ns	ns	ns	ns	ns

Note: *, ** and *** indicate significance levels at $p < 0.05$, $p < 0.01$ and $p < 0.001$, respectively; ns denotes no significant.

Table S3. Two-way analysis of variance for leaf water potential (ψ_l), leaf area (*LA*) and special leaf area (*SLA*) of potato plants grown under different vapor pressure deficit (VPD1, normal temperature and high relative humidity; VPD2, high temperature and relative humidity; VPD3, normal temperature and low relative humidity; VPD4, high temperature and low relative humidity) and irrigation (Irr, full or nill) at different harvest times.

Factor	Ψ_l (MPa)				LA (cm^2)				SLA (cm^2 g^{-1})			
	Harvest times				Harvest times				Harvest times			
	1	2	3	4	1	2	3	4	1	2	3	4
[Irr]	**	***	**	***	*	***	***	*	*	*	**	***
[VPD]	*	**	*	**	**	*	*	***	ns	ns	ns	ns
[Irr]×[VPD]	ns	ns	ns	ns	*	ns	ns	ns	ns	ns	ns	ns

Note: *, ** and *** indicate significance levels at $p < 0.05$, $p < 0.01$ and $p < 0.001$, respectively; ns denotes no significant.
